# A Rare Presentation of Epiploic Appendagitis as Chest Pain: A Case Report

**DOI:** 10.7759/cureus.61987

**Published:** 2024-06-09

**Authors:** Thomas A Elimihele, Sachin Kumar, Ifelunwa M Osanakpo, Nkechi Akata

**Affiliations:** 1 Internal Medicine, Meharry Medical College, Nashville, USA; 2 Internal Medicine, Spartan Health Sciences University School of Medicine, Vieux Fort, LCA; 3 Internal Medicine, All Saints University, Chicago, USA

**Keywords:** right upper quadrant tenderness, atypical presentation, colon, appendagitis, primary epiploic appendagitis (pea)

## Abstract

Epiploic appendagitis (EA) is an ischemic infarction of an epiploic appendage due to torsion or spontaneous thrombosis of the central vein of an epiploic appendage. It is a rare but benign and self-limiting cause of abdominal pain that is often misdiagnosed. The typical presentation of EA is lower abdominal pain, but pain can also occur in other parts of the abdomen. Presentation outside of the abdomen is a rare occurrence. Our patient presented with chest pain, and it was only through physical examination that mild right upper quadrant tenderness led to the suspicion of an intra-abdominal pathology, which was then confirmed with imaging. The patient responded to conservative management. Our possible explanation for this occurrence includes the proximity of the inflamed appendage to organs associated with chest pain and the possibility that patients sometimes describe pain location inaccurately.

## Introduction

Epiploic appendages are tiny colonic outpouches filled with fat and covered by a serosa layer. They extend from the outer surface of the colon into the peritoneal cavity. The adult colon typically contains around 50 to 100 epiploic appendages [1]. They are 1-2 cm thick and 0.5-5 cm long [1,2].

Epiploic appendagitis (EA) is characterized by inflammation of the epiploic appendage, which can occur either as a primary inflammation or as a secondary response. Primary epiploic appendagitis (PEA) results from either the twisting of an epiploic appendage or the spontaneous formation of a blood clot in an appendageal vein, leading to ischemic or hemorrhagic infarction and inflammation [2]. Secondary EA can be triggered by adjacent inflammatory conditions, such as diverticulitis [1,2]. Research indicates that this frequently occurs between the ages 20 and 50 years, with a mean age of 40 years, and it affects male more than female [1]. 

The blood supply of an epiploic appendage comes from one or two small end arteries, which are branches of the colonic vasa recta, drained through a single vein with a narrow pedicle. Due to its elongated shape, freely movable structure, and restricted blood flow, the epiploic appendage is susceptible to twisting, leading to ischemic or hemorrhagic infarction. The most common locations affected are the sigmoid colon and the cecum [2].

The primary symptom of EA is abdominal pain, with pain in the right and left lower quadrants being the most common locations. The pain may be aggravated by movement. Some patients also present with anorexia, nausea, vomiting, and diarrhea. Physical examination usually reveals localized abdominal tenderness with or without rebound tenderness. Laboratory results may also reveal leukocytosis [1,2].

Computed tomography (CT) scans or ultrasonography (USS) are used to diagnose EA. However, diagnosing EA solely through USS may be challenging as the accuracy is dependent on the experience of the operator. Given their availability and less dependence on the operator's experience, CT scans are fast becoming the preferred imaging modality in the United States and are almost always required for confirmation.

The mainstay treatment for EA is the use of nonsteroidal anti-inflammatory drugs although some cases are self-limiting. Antibiotics and surgical excision are rarely needed [1-4]. Here, we report a case of PEA with an atypical presentation of right-sided chest pain and right upper quadrant tenderness.

## Case presentation

A 56-year-old African American female presented to the emergency department (ED) with right-sided chest pain with associated shortness of breath of 24-hour duration. Pain was described as a constant dull pain on the right side of the chest, rated as 5/10, non-radiating, worse with breathing, with no known relieving factor. The patient endorsed having a fever of 101°F on home measurement, but the fever was not present at the ED. On review of the system, the patient denied any other respiratory, cardiovascular, or gastrointestinal symptoms. The examination, however, was notable for a female patient in mild painful distress, with mild tenderness in the right upper quadrant. The rest of the abdominal examination was normal, and there was no significant finding on the respiratory and cardiovascular examination and the other systems.

**Figure 1:**
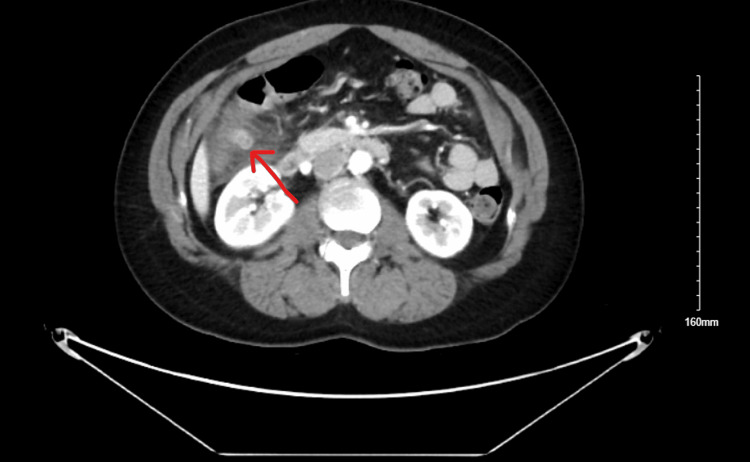
Abdominal CT showing a 14 mm oval, ill-defined soft tissue density near the hepatic flexure with adjacent inflammatory changes in the fat consistent with epiploic appendagitis (red arrow) CT: Computed tomography

The patient's laboratory result showed normal leukocyte count with mild anemia, comprehensive metabolic panel (CMP) was unremarkable, troponin I ultra was <0.01 (reference range: 0-0.034), D. dimer was 0.41 (reference range: 0-0.5), and she had a negative COVID test. Electrocardiography (EKG) showed sinus rhythm with nonspecific t-wave abnormalities but no acute ST changes. The CT of the chest was normal. However, abdominal CT revealed a 14 mm oval, somewhat ill-defined soft tissue density near the hepatic flexure with adjacent inflammatory changes in the fat consistent with EA as shown in Figure 1.

The patient was given supportive treatment with ketorolac for pain management and intravenous fluid. The patient had received a dose of piperacillin-tazobactam at the ED, which was discontinued. She was admitted to the ward for observation with plans for surgical evaluation if there was no resolution of symptoms, given the report of fever at home and also the need to trend the patient's troponin since her presenting symptom was chest pain, but troponin remained unremarkable. The patient responded well to conservative management and was discharged home the following day.

## Discussion

Epiploic appendages, which emerge from the exterior of the colon, ranging from the cecum to the rectosigmoid area, are peritoneal pouches tethered by vascular stalks. Typically, individuals possess approximately 100 of these appendages, comprising adipose tissue and blood vessels, with lengths varying between 0.5 and 5 cm [5].

The primary common location of EA is the lower quadrants of the abdomen, with the most frequent presentation occurring in the left lower quadrant, often misdiagnosed as diverticulitis. In a study comprising 58 patients diagnosed with EA, 48% of cases were located in the sigmoid colon, 28% in the descending colon, 7% in the transverse colon, and 17% in the ascending colon [6,7].

Another study reported that the majority occur in the rectosigmoid (57%) or ileocecum (26%), which accounts for their common mimic of lower abdominal pain. Rarer occurrences occur in the ascending (9%), transverse (6%), and descending colon (2%) [8,9].

Therefore, a typical presentation of EA manifests as sudden, acute-onset abdominal pain, typically localized to the lower quadrants and occasionally accompanied by fever, but not commonly. Physical examination may reveal tenderness and possible guarding. The condition often poses diagnostic challenges due to its resemblance to the surgical abdomen, necessitating a careful approach to management [2]. Presentation with an extra-abdominal symptom is not a common occurrence.

Our patient had PEA, which was prone to a heightened chance of misdiagnosis due to complaints of chest pain, associated shortness of breath, and a subjective fever. The patient's manifestation of chest discomfort exacerbated by respiration posed a diagnostic challenge, mirroring presentations commonly associated with conditions such as pulmonary embolism, myocardial infarction, or lower lobe pneumonia. On the contrary, investigative modalities, including D-dimer assay, troponin levels, chest radiography, and CT imaging, revealed no discernible thoracic abnormalities. It was the identification of a subtle right upper quadrant tenderness during physical examination that prompted consideration of an intra-abdominal pathology. This informed the decision to include abdominal imaging among the investigative studies, leading to the diagnosis of PEA, localized to the right hepatic flexure, which in itself is a rare location as most cases are located in the lower abdomen, as stated above. 

Notably, this presentation bore a resemblance to acute cholecystitis, typified by right upper quadrant pain and chest discomfort exacerbated by breathing. A plausible explanation for this atypical presentation may be attributed to the close anatomical proximity of inflamed hepatic flexure epiploic appendages to the gallbladder, engendering local irritation of somatic nerve fibers, thus precipitating referred pain. Several corroborative case reports substantiate the hypothesis that EA localized to the hepatic flexure can mimic acute cholecystitis, characterized by right upper quadrant pain and a positive Murphy sign [6,8-9]. In these documented instances, imaging studies revealed inflamed appendages juxtaposed with noninflamed gallbladders, thereby accentuating the clinical challenge of distinguishing between these two pathologies, but this can be easily distinguished with the help of imaging modalities such as CT and USS [7,9]. 

Another critical factor to consider is that patients often inaccurately describe their pain locations. These spatial inaccuracies have been well-documented in the literature. In a study by Gordon et al. to assess the accuracy of a patient's unassisted pain drawing, an accurate representation was only made in about 43% of instances [10]. This could also explain our patients' misrepresentation of abdominal pain. 

## Conclusions

Our case report draws attention to an atypical presentation of PEA; this is particularly important to note, given that cases of PEA are often misdiagnosed with other causes of acute abdomen. This patient, in addition to having a PEA located in the hepatic flexure, a relatively uncommon location for it, also presented with chest pain instead of abdominal symptoms, which is often the case. Both situations increased the chances of being misdiagnosed. 

The fact that a mild right upper quadrant tenderness led to the inclusion of abdominal imaging underscores the need for a thorough physical exam just as much as a detailed history, as patients may be inaccurate in describing the location of their symptoms even if the history taken was detailed.
